# Supplementary material for: Preparation and Characterization of Natural Silk Fibroin Hydrogel for Protein Drug Delivery
Source: Molecules. 2022 May 25;27(11):3418. doi: 10.3390/molecules27113418 (PMC9181960; doi:10.3390/molecules27113418)
Supplement: Supplementary file 1 [file molecules-27-03418-s001.zip › molecules-1724924-supplementary.pdf]

molecules

Supplementary Materials

Preparation and Characterization of Natural Silk Fibroin Hydrogel for Protein Drug Delivery

Junwei Liu [1,2,3], Haowen Sun [2], Yuwei Peng [2], Ligen Chen [2,3], Wei Xu [2,3] and Rong Shao [1,3,*]

1 School of Chemistry and Chemical Engineering, Yancheng Institute of Technology, Yancheng 224051, China; liujunwei_1222@163.com
2 School of Marine and Bioengineering, Yancheng Institute of Technology, Yancheng 224051, China; shw987052988@163.com (H.S.); pengyvwei1114@163.com (Y.P.); ycit549638894@outlook.com (L.C.); xuweiyc@163.com (W.X.)
3 Jiangsu Key Laboratory of Biochemistry and Biotechnology of Marine Wetland, Yancheng Institute of Technology, Yancheng 224051, China
* Correspondence: sr@ycit.cn

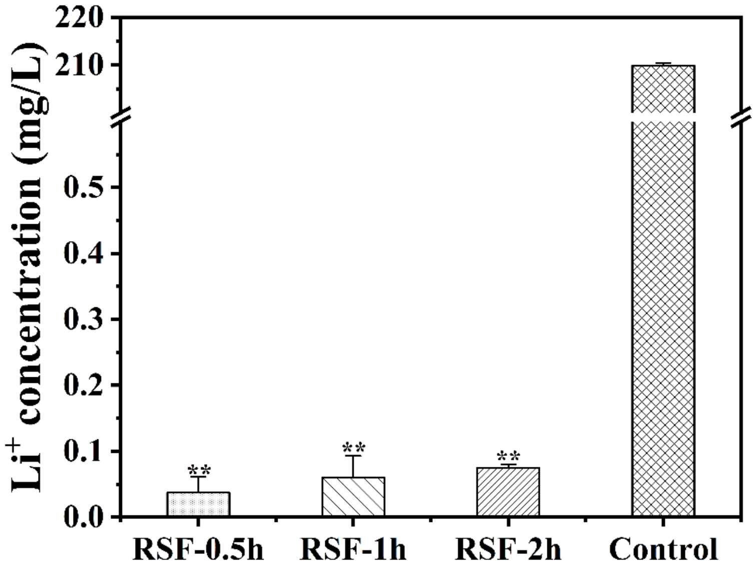

Figure S1. Li^+ content in silk fibroin aqueous solution after dialysis. Compared with the control group, "**" means extremely significant difference, $p < 0.01$.

According to the ICP test results, the Li^+ contents in the three silk fibroin aqueous solutions were 0.037 ± 0.024, 0.060 ± 0.033, and 0.075 ± 0.005 mg/L, respectively, compared with the lithium element content in the control group of 209.89 ± 0.48 mg/L, which were all very low concentration levels ($p < 0.01$).

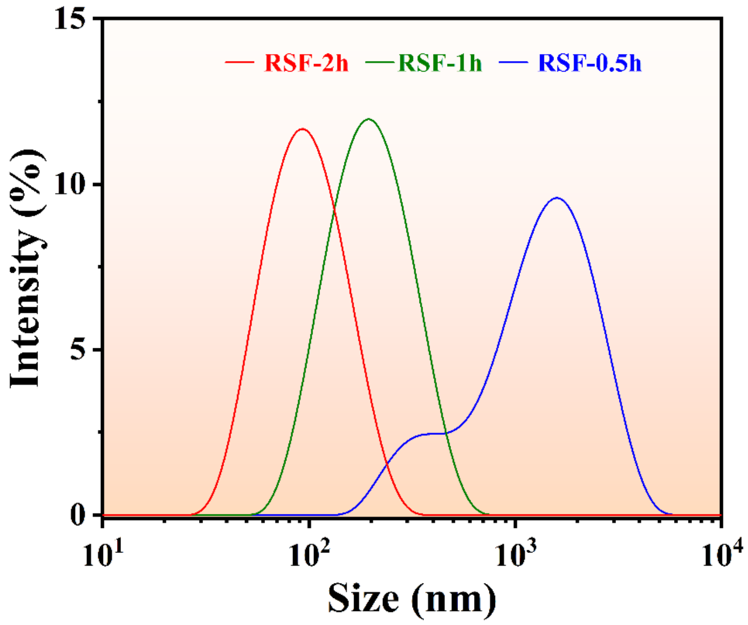

Figure S2. Particle size distribution of silk fibroin in aqueous solutions with different molecular weights.

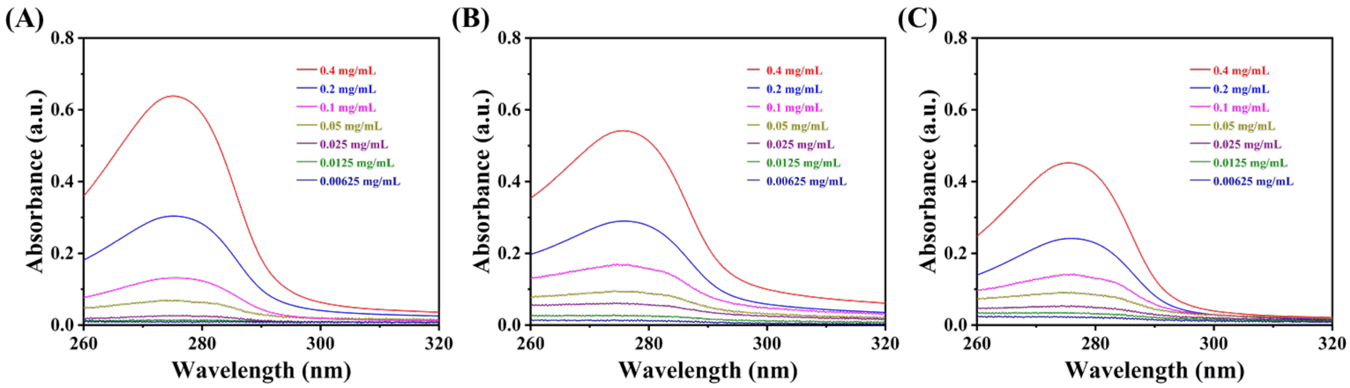

Figure S3. UV spectra of silk fibroin aqueous solutions with different molecular weights. (**A**) RSF-0.5h; (**B**) RSF-1h; (**C**) RSF-2h.

Table S1. Contents of organic elements in the freeze-dried samples.

**Freeze-Dried Samples**		**Element Mass Fraction / %**			**Element Mass Ratio**	
		**C**	**N**	**H**	**C/N**	**C/H**
Silk fibroin aqueous solution	RSF-0.5h	17.27 ± 0.13	44.66 ± 0.24	6.35 ± 0.05	2.59	7.03
	RSF-1h	17.14 ± 0.09	44.99 ± 0.31	6.42 ± 0.08	2.62	7.01
	RSF-2h	17.53 ± 0.11	45.07 ± 0.37	6.37 ± 0.06	2.57	7.08
Silk fibroin hydrogel	RSF-0.5h	17.20 ± 0.11	44.72 ± 0.31	6.22 ± 0.04	2.60	7.19
	RSF-1h	17.62 ± 0.17	45.65 ± 0.39	6.30 ± 0.06	2.59	7.25
	RSF-2h	17.78 ± 0.14	45.93 ± 0.42	6.30 ± 0.06	2.58	7.29

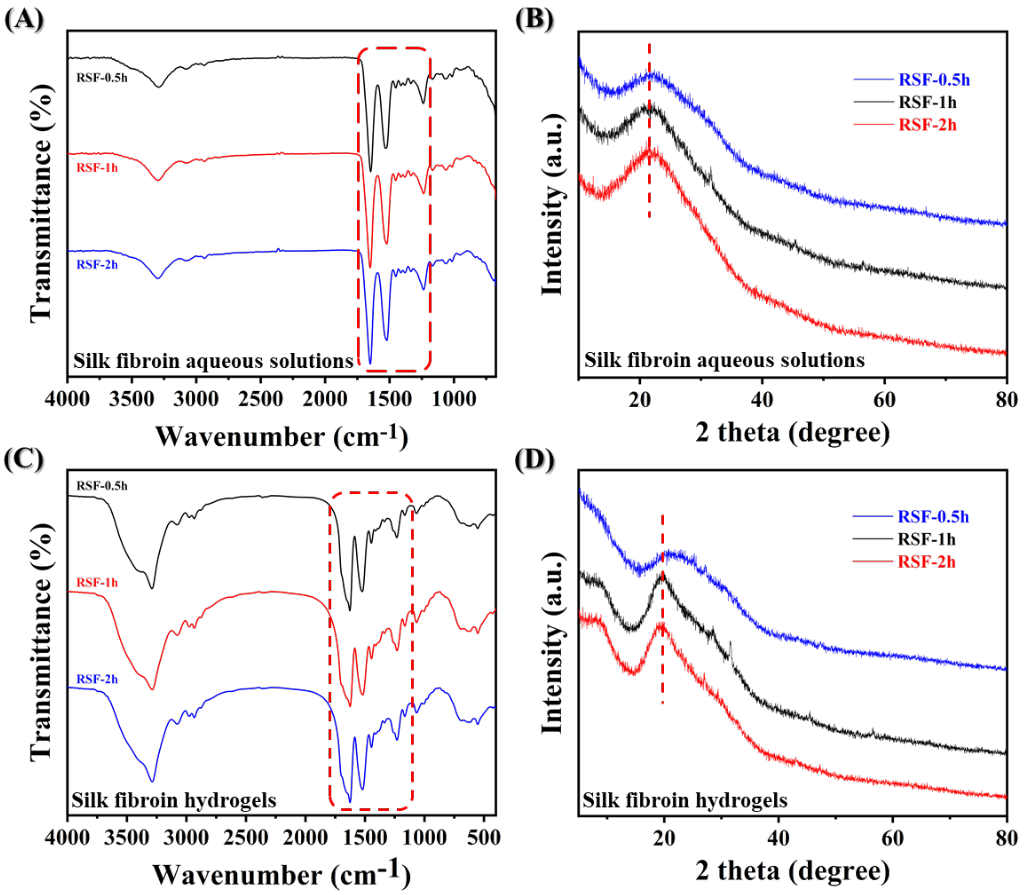

Figure S4. Aggregated structure of silk fibroin. FT-IR (**A**) and XRD (**B**) spectra of silk fibroin aqueous solutions with different molecular weights. FT-IR (**C**) and XRD (**D**) spectra of silk fibroin hydrogels with different molecular weights.

The main functional group changes of silk fibroin obtained under different degumming times were investigated by FT-IR, and the influence of degumming time on the molecular conformation of silk fibroin was evaluated to a certain extent. The conformations, such as β-sheet, α-helix, random coil, and β-turn, of silk fibroin molecular chains correspond to different absorption peak positions in the infrared absorption spectrum [46], and the results are shown in Figure S4(A,C). The infrared spectra of silk fibroin samples with different molecular weights were basically the same, and the characteristic absorption peaks around 3300 cm^{-1} and 3290 cm^{-1} were mainly attributed to the stretching vibration of –OH in the silk fibroin structure [47]. It can be seen from Figure S4(A) that RSF-0.5h had strong characteristic absorption peaks at 1646 cm^{-1} (amide I), 1532 cm^{-1} (amide II), and 1236 cm^{-1} (amide III). Amides I and II correspond to the α-helical conformation, and amide III corresponds to the random coil conformation. RSF-1h had strong characteristic absorption peaks at 1650 cm^{-1} (amide I), 1522 cm^{-1} (amide II), and 1236 cm^{-1} (amide III). Amide I corresponds to the α-helix conformation, amide II corresponds to the β-sheet conformation, and amide III corresponds to the random coil conformation. RSF-2h had characteristic absorption peaks at 1649 cm^{-1} (amide I), 1521 cm^{-1} (amide II), and 1236 cm^{-1} (amide III). Amide I corresponds to the α-helical conformation, amide II corresponds to the β-sheet conformation, and amide III corresponds to the random coil conformation. According to the protein secondary structure analysis of amides I, II ,and III, silk fibroin mainly exists in α-helix and random coil conformations in aqueous solution [48]. It can be seen in Figure S4(C) that the lyophilized samples of silk fibroin hydrogels with three molecular weights had a strong characteristic absorption peaks at around 1625 cm^{-1}, 1520 cm^{-1}, and 1230 cm^{-1}, respectively. The peak shape was sharp, corresponding to the absorption peaks of silk fibroin amide I, amide II, and amide III, respectively, showing a typical

β-sheet conformation in which amide III corresponds to a mixed conformation of β-sheets and random coils. According to the above protein secondary structure analysis of amides I, II, and III, it can be seen that the silk fibroin in hydrogel mainly existed in the form of β-sheets, which mainly depended on the internal molecular chains of the silk fibroin. The crystalline regions formed by hydrogen bonds acted as physical crosslinks, which slipped during gel formation and freeze-drying and clumped together to form a β-sheet conformation [49]. Therefore, the silk fibroin in the aqueous solution belonged to the typical silk I structure, and the extension of the degumming time may have caused the silk fibroin structure to change from silk I to silk II; the silk fibroin in the hydrogel belonged to the typical silk II structure since the hydrogen bonds between the molecular chains in the silk II structure were difficult to break. The performance of the silk fibroin hydrogels was stable and insoluble in water [50].

The position of the diffraction peak can reflect the related crystal structure of the silk fibroin material, and the intensity and width of the diffraction peak directly reflect the degree of crystallinity of the silk fibroin material [51]. According to previous reports, in the XRD pattern of silk fibroin, 12.2°, 19.7°, 24.7°, 28.2°, 32.3°, 36.8°, and 40.1° are the main diffraction absorption peaks of the silk I structure (α-helix and random coil), and 9.1°, 18.9°, 20.7°, 24.3°, and 39.7° are the main diffraction absorption peaks of the silk II structure (β-sheet) [52]. The crystal structures of silk fibroin samples of three molecular weights were analyzed by an X-ray diffractometer. It can be seen in Figure S4(B) that the three silk fibroin samples in the aqueous solution only had a "mound peak" with a large span in the range of 19.7°-24.7°, indicating that the silk fibroin in the aqueous solution lacked regular crystals. The region was dominated by a silk I structure (α-helix and random coil); that is, the "mound peak" peak near 22° could be identified as the non-crystalline region of the silk fibroin. With the extension of degumming time, the peak-shaped structure changed from "short and wide" to "sharp and narrow", which was related to the change in the internal crystallinity of the silk fibroin, and the destruction of the amorphous structure resulted in the formation of crystalline regions. It can be seen in Figure S4(D) that the silk fibroin sample had a weak diffraction peak near 9.1° and an obvious diffraction peak near 19.7°, indicating that there were two crystal structures of silk I (α-helix and random coil) and silk II (β-sheet) in the silk fibroin hydrogel samples. It can be seen that some silk fibroin molecular conformations transitioned from an amorphous structure, or a silk I (α-helix and random coil) structure, to a Silk II (β-sheet) crystalline structure, which is consistent with the results of the infrared spectroscopy [53].

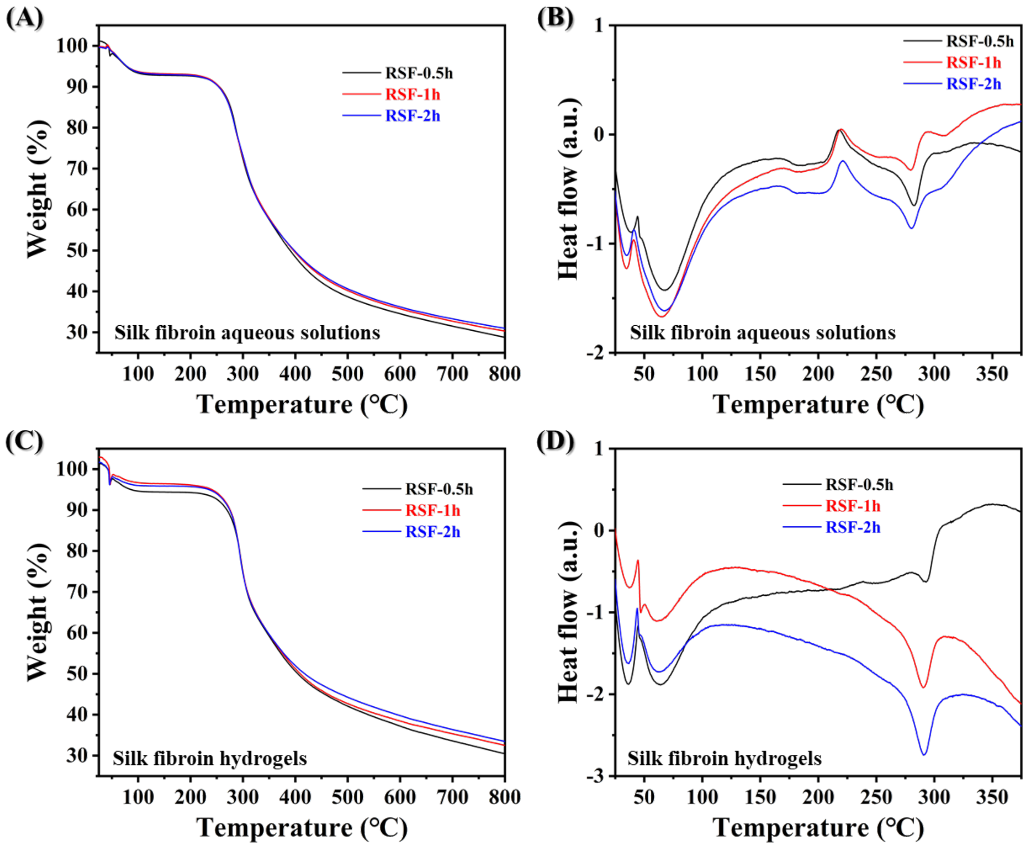

Figure S5. Thermal stability analysis of silk fibroin. TGA (**A**) and DSC (**B**) diagrams of silk fibroin aqueous solutions with different molecular weights. TGA (**C**) and DSC (**D**) diagrams of silk fibroin hydrogels with different molecular weights.

The thermal stability of the three silk fibroin aqueous solution freeze-dried products of RSF-0.5h, RSF-1h, and RSF-2h were analyzed and determined, and the results are shown in Figure S5(A,B). It can be seen in Figure S5(A) that the first stage of weight loss occurred in the silk fibroin samples in the range of 50–100 °C, which was mainly due to the loss of free water and bound water in the silk fibroin samples [54]. The weight loss of the three silk fibroin samples in the second stage occurred at 280–400 °C, which was the process of thermal degradation of silk fibroin, and the mass loss of the silk fibroin samples at this stage was about 50%. In addition, the thermal degradation residues of RSF-0.5h, RSF-1h, and RSF-2h at 800 °C were 28.78%, 30.31%, and 30.98%, respectively, indicating that with the increase in the crystallinity of the silk fibroin samples, the residual amount of thermal degradation increased gradually because the prolongation of sodium carbonate treatment time helped to improve the thermal stability of the silk fibroin samples. The DSC curves of the three molecular weight silk fibroin lyophilized samples are shown in Figure S5(B). From the figure, it can be clearly observed that RSF-0.5h, RSF-1h, and RSF-2h had two endothermic peaks and one exothermic peak. The DSC curves of the three tended to be consistent, and the endothermic peak positions were all around 70 °C and 280 °C. The temperature around 70 °C indicates the evaporation of free water in the silk fibroin sample, and the mass loss of the sample was about 10% in this process because the free water was mainly concentrated in the amorphous region of the silk fibroin molecule. However, the amorphous regions of RSF-0.5h, RSF-1h, and RSF-2h were almost the same, resulting in the same mass loss of the three at this stage. As the temperature continued to increase, an exothermic peak appeared around 220 °C, indicating that the silk fibroin was further crystallized. In this process, the internal α-helix and random coil conformation of the lyophilized silk fibroin aqueous solution changed to a more stable β-sheet conformation with the continuous increase in temperature, thereby releasing heat to form an

exothermic peak. With the further increase in temperature, the molecular chain of silk fibroin gradually broke from the non-crystalline region to the crystalline region, and the thermal decomposition peak occurred around 280 °C due to the low crystallinity of the β-sheet conformation of silk fibroin. In summary, there was a large number of α-helix and random coil conformations in the silk fibroin aqueous solution, mainly in the silk I structure, and the thermal properties of the lyophilized silk fibroin aqueous solution obtained at different degumming times were basically the same; that is, the degumming time had no significant effect on the thermodynamic properties of the obtained silk fibroin aqueous solutions.

From the TGA curve in Figure S5(C), it can be seen that the first stage of weight loss in silk fibroin samples in the temperature range of 50–100 °C was due to the loss of water in the samples as the temperature increased [54]. The weight loss of the three silk fibroin samples in the second stage all occurred at 280–400 °C, which was mainly caused by the thermal degradation caused by the breakage of internal peptide bonds and amino acid side chain groups of the silk fibroin [55]. The thermal degradation residues of the three types of silk fibroin were 30.46%, 32.51%, and 33.47%, respectively. The results showed that the degradation residues of the silk fibroin samples increased with the extension of the degumming time within 2 h, indicating that the extension of degumming time can make silk fibroin samples have higher thermal stability. It can be seen from the DSC curve of Figure S5(D) that the overall trend of the three silk fibroin samples was similar, and there were two obvious endothermic peaks around 65 °C and 290 °C, respectively. The temperature around 65 °C indicates the evaporation of free water in the silk fibroin sample. The mass loss of the three in this stage was equivalent, and the mass loss of the sample was about 10%. When the temperature reached around 290 °C, an obvious endothermic peak appeared, indicating that the silk fibroin molecular chain began to break from the amorphous region to the crystalline region, and the β-sheet conformation of silk fibroin with low crystallinity was gradually decomposed by heat. In addition, comparing the DSC curve of the silk fibroin aqueous solution, it can be found that there was no obvious exothermic peak in the DSC curve of the silk fibroin hydrogel, which further indicates that the interior of the silk fibroin hydrogel sample mainly existed in a β-sheet conformation [56].

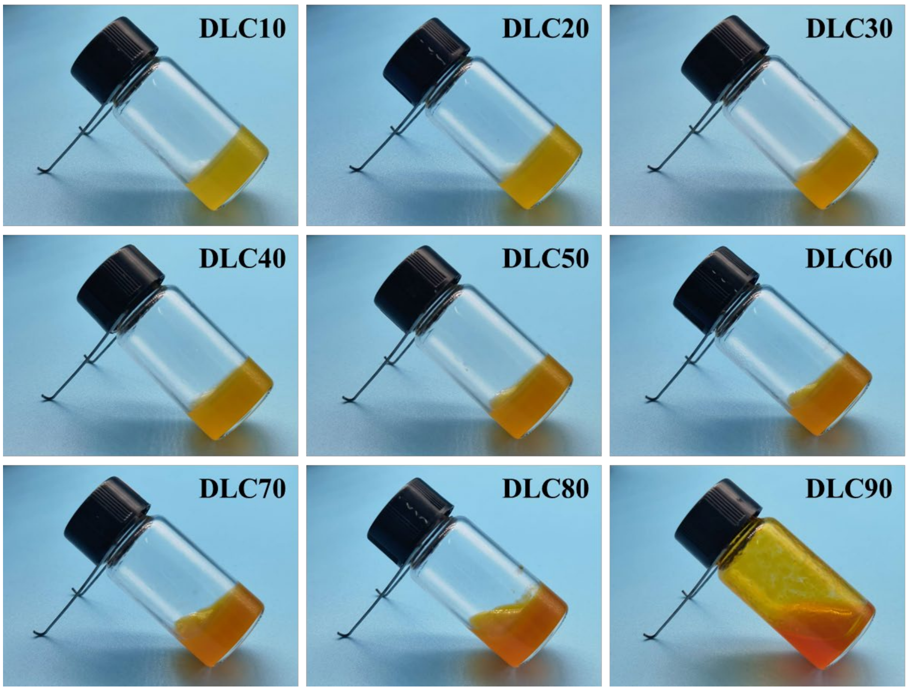

Figure S6. Digital photographs of RSF10 drug-containing hydrogels with different drug loadings.

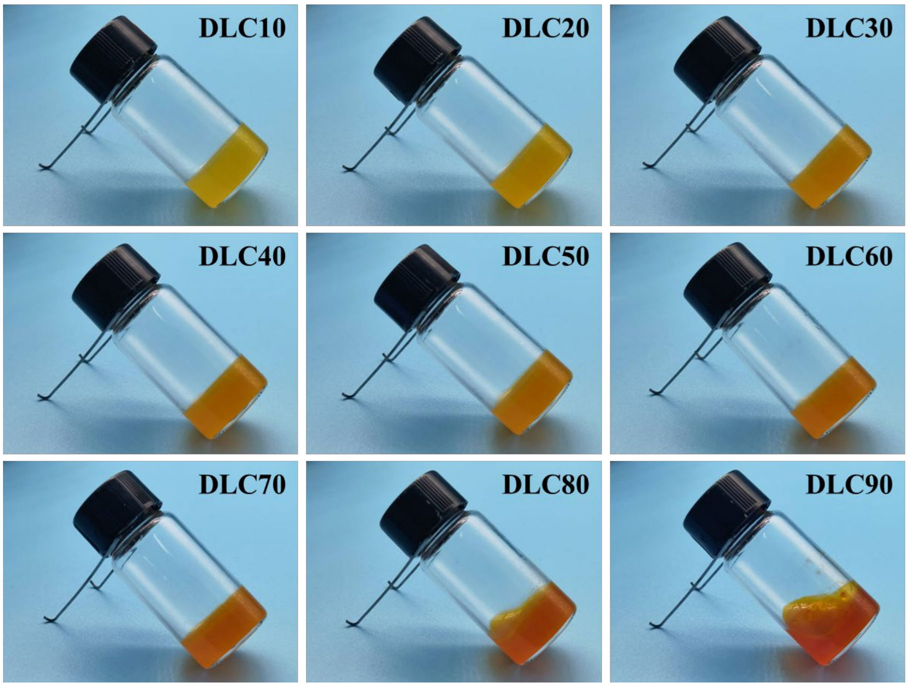

Figure S7. Digital photographs of RSF15 drug-containing hydrogels with different drug loadings.

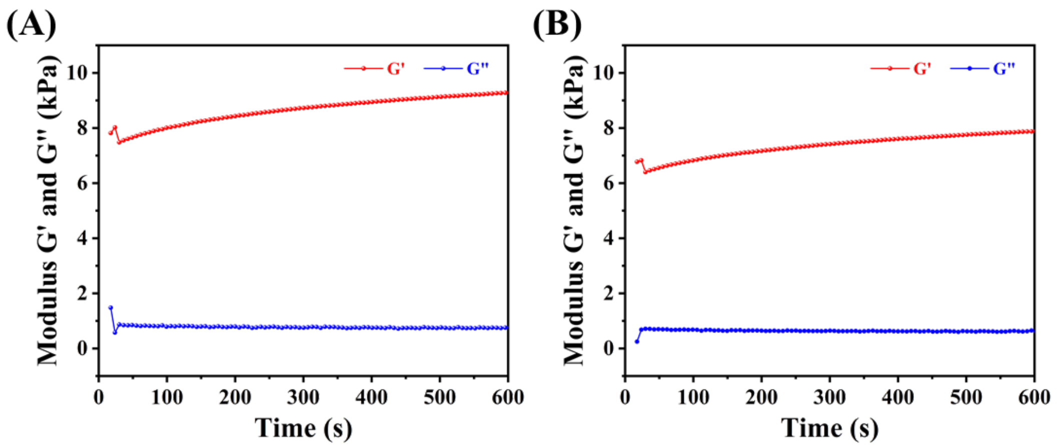

Figure S8. Rheological properties of drug-containing hydrogels (**A**) DLC40 and (**B**) DLC20.

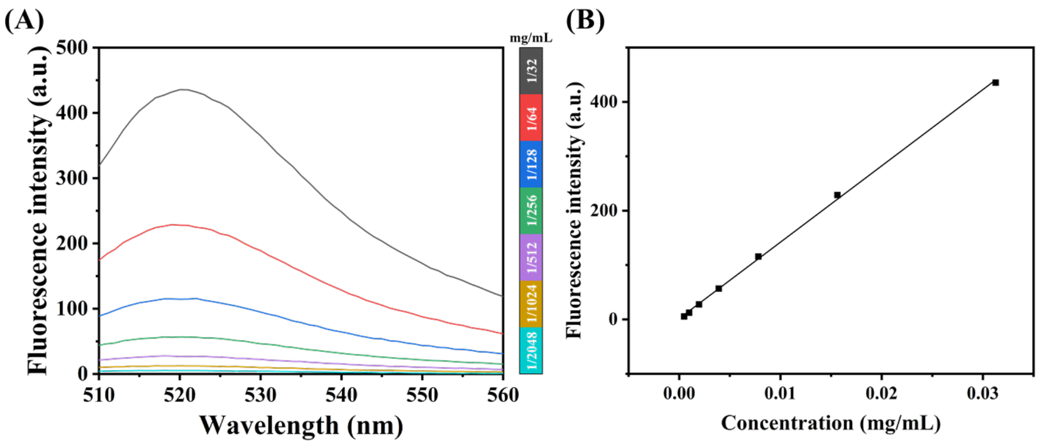

Figure S9. Fluorescence spectrum (**A**) and standard curve (**B**) of BSA-FITC.

A certain amount of BSA-FITC was dissolved in PBS buffer to prepare a BSA-FITC standard solution with a concentration of 1 mg/mL. The standard stock solution was serially diluted with PBS buffer until the concentration was 2^{-11} mg/mL, and the fluorescence spectrum of the standard solution was collected by a JASCO FP-6500 fluorescence spectrometer. The excitation wavelength was 490 nm, and the scan range was 510–560 nm. Taking the fluorescence intensity (a.u.) as the ordinate and the concentration of BSA-FITC solution (mg/mL) as the abscissa to draw a standard curve, the following linear regression equation is obtained:

$$FI = 14035C + 1.7316\ (R^2 = 0.9992)$$

where, FI is the fluorescence intensity, a.u.; C is the solution concentration, mg/mL; and R^2 is the linear regression coefficient.

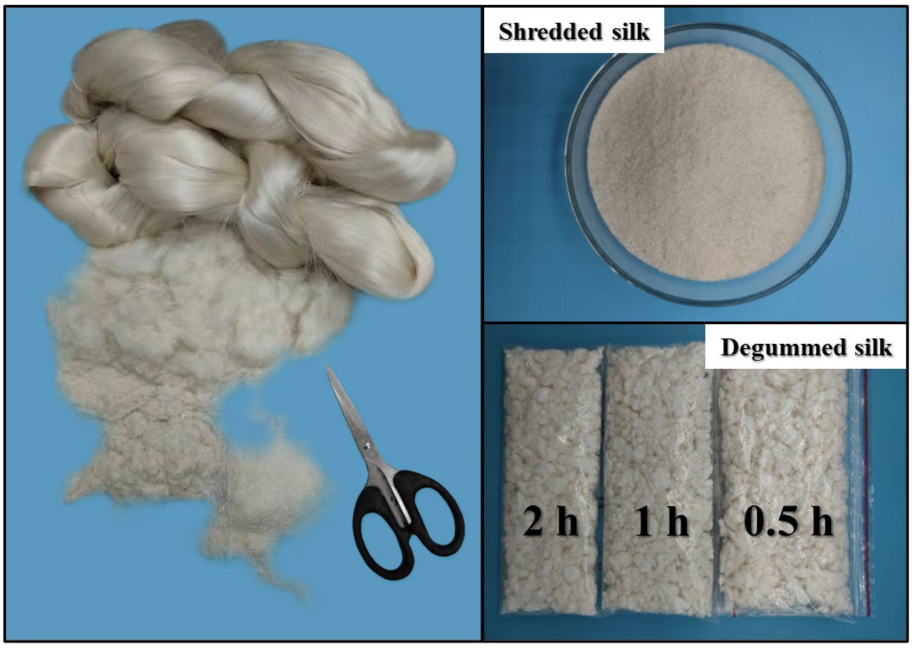

Figure S10. Acquisition of shredded silk and degummed silk sample diagram.

Degumming of Natural Silk

Raw silk textile yarns (derived from mulberry cocoons) were cut into pieces with scissors, and 10.0 g of shredded silk was accurately weighed. The silk was poured into a 0.02 mol/L sodium carbonate aqueous solution and stirred evenly to fully infiltrate the shredded silk. The ratio of material to liquid was 1:10. The degumming treatment was carried out by stirring and boiling in sodium carbonate solution, repeated twice. After filtration, it was immersed in warm water four times and then rinsed four times with ultrapure water to remove the sodium carbonate and sericin remaining on the degummed silk. After filtration, a picric acid-carmine indicator was used to check whether the sericin was completely removed. Finally, the degummed silk was spread on a glass plate and placed in an oven. The temperature was set to 60 °C, and it was dried at a constant temperature for 12 h. After being taken out, the degummed silk was obtained by pulling and loosening and could be dried and stored for later use. The silk fibroin extracted under the degumming times of 0.5, 1, and 2 h were named RSF-0.5h, RSF-1h, and RSF-2h, respectively, the same below (Figure S10).

Preparation and concentration determination of silk fibroin aqueous solution

Four dry and clean small 100 mL beakers were prepared. Next, 2.0 g of dry degummed silk was weighed in each small beaker and it was dissolved in 20 mL of 9.3 mol/L lithium bromide solution. The ratio of material to liquid was 1:10. It was placed in a constant temperature water bath at 60 °C to react for 4 h, taken out to cool, and poured into an activated dialysis bag with a length of 50 cm and a molecular weight cut-off of 3500 Da. Both ends were clamped with sealing clips, and it was placed in deionized water for dialysis for 3 days (water was changed every 2 h in the early stage, and water is changed every 6 h in the middle and late stage). After the dialysis was completed, flocculent aggregates and small particles of impurities were removed by centrifugation or filtration, and the silk fibroin aqueous solution with a concentration of 30 mg/mL was finally obtained, which was a pale yellow transparent liquid. The preparation process of silk fibroin aqueous solution is shown in Scheme 1B.

Preparation of Fluorescently Labeled BSA

According to the characteristic that the isothiocyanate group (–N=C=S) of fluorescein isothiocyanate isomer I (FITC) can interact with the free amino group (–NH_2) on BSA amino acid, BSA was labeled with FITC. A certain mass of BSA was weighed into a 250 mL round-bottomed flask, an appropriate amount of ultrapure water was added, and it was shaken up to a concentration of 10 mg/mL. A small amount of FITC was weighed and dissolved in DMF to obtain a 20 mg/mL FITC solution. The FITC solution was slowly dropped into the BSA solution at a volume ratio of 1:100. The round-bottomed flask was placed on a magnetic stirrer to react for 48 h under dark conditions, and the obtained reaction product was poured into a dialysis bag (molecular weight cut-off of 3500 Da), and dialyzed with deionized water for 3 days in the dark to obtain a purified BSA-FITC solution, freeze-dried at −60 °C, and further dried in a vacuum desiccator to obtain fluorescently labeled BSA drugs, which were stored in a refrigerator at 4 °C in the dark for future use.

Degradation in Different Media

In this work, the degradation behavior of two concentrations of silk fibroin hydrogels (RSF15 and RSF10) in six groups of media was explored.

1. PB media: PB buffers with pH 5.0, pH 7.0, pH 7.4, and pH 8.0;
2. Hydrochloric acid media: 0.1, 0.5, 1, and 2 mol/L HCl solutions;
3. Sodium hydroxide media: 0.1, 0.2, 0.5, and 1 mol/L NaOH solutions;
4. Organic solvent media: Ethanol, DMF, and DMSO;
5. Enzyme media: PBS (control), elastase (10 U/mL), and GSH (1 mg/mL);
6. NaCl media: 0, 0.2, 1, and 2 mol/L NaCl solutions.

References

46. Dong, A.C.; Prestrelski, S.J.; Allison, S.D.; Carpenter, J.F. Infrared spectroscopic studies of lyophilization-and temperature-induced protein aggregation. *J. Pharm. Sci.* **1995**, *84*, 415–424.
47. Dong, Z.F.; Wang, Q.; Du, Y.M. Alginate/gelatin blend films and their properties for drug controlled release. *J. Membr. Sci.* **2006**, *280*, 37–44.
48. Panjapheree, K.; Kamonmattayakul, S.; Meesane, J. Biphasic scaffolds of silk fibroin film affixed to silk fibroin/chitosan sponge based on surgical design for cartilage defect in osteoarthritis. *Mater. Des.* **2018**, *141*, 323–332.
49. Qi, Y.; Wang, H.; Wei, K.; Yang, Y.; Zheng, R.Y.; Kim, I.S.; Zhang, K.Q. A review of structure construction of silk fibroin biomaterials from single structures to multi-level structures. *Int. J. Mol. Sci.* **2017**, *18*, 237–248.
50. Lopes, L.M.; Moraes, M.A.; Beppu, M.M. Phase diagram and estimation of flory-huggins parameter of interaction of silk fibroin/sodium alginate blends. *Front. Bioeng. Biotech.* **2020**, *8*, 2296–2385.
51. Devi, D.; Sarma, N.S.; Talukdar, B.; Chetri, P.; Baruah, K.C.; Dass, N.N. Study of the structure of degummed Antheraea assamensis (muga) silk fibre. *J. Text. Inst.* **2011**, *102*, 527–533.
52. Ming, J.F.; Li, M.M.; Han, Y.H.; Chen, Y.; Li, H.; Zuo, B.Q.; Pan, F.K. Novel two-step method to form silk fibroin fibrous hydrogel. *Mat. Sci. Eng. C-Mater.* **2016**, *59*, 185–192.
53. Barud, H.G.O.; Barud, H.S.; Cavicchioli, M. Preparation and characterization of a bacterial cellulose/silk fibroin sponge scaffold for tissue regeneration. *Carbohyd. Polym.* **2015**, *128*, 41–51.
54. Samal, S.K.; Dash, M.; Chiellini. F.; Wang, X.Q.; Chiellini, E.; Declercq, H.A.; Kaplan, D.L. Silk/chitosan biohybrid hydrogels and scaffolds via green technology. *RSC Adv.* **2014**, *4*, 53547–53556.
55. Nogueira, G.M.; Weska, R.F.; Vieira, W.C.; Polakiewicz, B.; Rodas, A.C.D.; Higa, O.Z.; Beppu, M.M. A new method to prepare porous silk fibroin membranes suitable for tissue scaffolding applications. *J. Appl. Polym. Sci.* **2009**, *114*, 617–623.
56. Yucel, T.; Cebe, P.; Kaplan. D.L. Vortex-induced injectable silk fibroin hydrogels. *Biophys. J.* **2009**, *97*, 2044–2050.
